# Antioxidants and NOX1/NOX4 inhibition blocks TGFβ1-induced CCN2 and α-SMA expression in dermal and gingival fibroblasts

**DOI:** 10.1371/journal.pone.0186740

**Published:** 2017-10-19

**Authors:** Hannah Murphy-Marshman, Katherine Quensel, Xu Shi-wen, Rebecca Barnfield, Jacalyn Kelly, Alex Peidl, Richard J. Stratton, Andrew Leask

**Affiliations:** 1 Department of Dentistry, Schulich School of Medicine and Dentistry, University of Western Ontario, London, Ontario, Canada; 2 Centre for Rheumatology, Royal Free and University College Medical School, London, United Kingdom; 3 Department of Biology, Schulich School of Medicine and Dentistry, University of Western Ontario, London, Ontario, Canada; 4 Department of Physiology and Pharmacology, Schulich School of Medicine and Dentistry, University of Western Ontario, London, Ontario, Canada; Medical University of South Carolina, UNITED STATES

## Abstract

TGFbeta induces fibrogenic responses in fibroblasts. Reactive oxygen species (ROS)/nicotinamide adenine dinucleotide phosphate (NADPH) oxidase (NOX) may contribute to fibrogenic responses. Here, we examine if the antioxidant N-acetylcysteine (NAC), the NOX inhibitor diphenyleneiodonium (DPI) and the selective NOX1/NOX4 inhibitor GKT-137831 impairs the ability of TGFbeta to induce profibrotic gene expression in human gingival (HGF) and dermal (HDF) fibroblasts. We also assess if GKT-137831 can block the persistent fibrotic phenotype of lesional scleroderma (SSc) fibroblasts. We use real-time polymerase chain reaction and Western blot analysis to evaluate whether NAC and DPI impair the ability of TGFbeta1 to induce expression of fibrogenic genes in fibroblasts. The effects of GKT-137831 on TGFbeta-induced protein expression and the persistent fibrotic phenotype of lesional scleroderma (SSc) fibroblasts were tested using Western blot and collagen gel contraction analyses. In HDF and HGF, TGFbeta1 induces CCN2, CCN1, endothelin-1 and alpha-smooth muscle actin (SMA) in a fashion sensitive to NAC. Induction of COL1A1 mRNA was unaffected. Similar results were seen with DPI. NAC and DPI impaired the ability of TGFbeta1 to induce protein expression of CCN2 and alpha-SMA in HDF and HGF. GKT-137831 impaired TGFbeta-induced CCN2 and alpha-SMA protein expression in HGF and HDF. In lesional SSc dermal fibroblasts, GKT-137831 reduced alpha-SMA and CCN2 protein overexpression and collagen gel contraction. These results are consistent with the hypothesis that antioxidants or NOX1/4 inhibition may be useful in blocking profibrotic effects of TGFbeta on dermal and gingival fibroblasts and warrant consideration for further development as potential antifibrotic agents.

## Introduction

Fibrotic conditions are characterized by the excessive deposition and remodeling of extracellular matrix by fibroblasts residing within connective tissue. There is no universally agreed-upon treatment for fibroproliferative conditions such as scleroderma (systemic sclerosis, SSc) or gingival hyperplasia, a condition which often occurs in response to anticonvulsant phenytoin, antihypertensive calcium channel blockers, and immunosuppressant cyclosporine therapy [1, 2]. Transforming growth factor (TGF)β has been implicated as a central mediator of fibrosis [3, 4]. Thus, developing methods of blocking TGFβ-driven fibrosis may result in therapeutically relevant antifibrotic strategies. Unlike in other adult tissues, fibrotic responses in the oral cavity do not elicit scar tissue deposition, but are instead characterized by gingival overgrowths, often in response to medications [2, 5]. Intriguingly, the molecular mechanisms underlying the induction of the profibrotic mediator CCN2/CTGF appear to differ between dermal and gingival fibroblasts in terms of a requirement for c-Jun kinase [6,7]. Compared with dermal fibroblasts, gingival fibroblasts appear to display a less potent response to both mechanical strain and TGFβ in terms of CCN2 induction [8]. As such, comparing the molecular mechanism underlying the signaling responses of dermal and gingival fibroblasts to fibrogenic stimuli, such as TGFβ, is of inherent value.

Increasing evidence, most notably in mesangial cells, has shown that TGFβ1 differentiates fibroblasts into a profibrotic myofibroblast phenotype via NADPH oxidase (NOX) homolog Nox4 and reactive oxygen species (ROS)-induced fibroblast and mesangial cell activation [9]. The antioxidant N-acetylcysteine (NAC) may have some benefit in treating idiopathic pulmonary fibrosis [10]. In addition, NAC alleviates the elevated contractile and migratory capability of lesional SSc dermal fibroblasts [11]. More recently, it was shown that NOX4 is overexpressed in lesional SSc fibroblasts [12]. GKT-137831, a newly identified inhibitor which specifically targets NOX1/4, is currently under clinical development [13]. Thus, antioxidants or GKT-137831 might impair the ability of TGFβ to induce expression of profibrotic genes in human dermal and gingival fibroblasts; however, this hypothesis has yet to be evaluated. In this report, we address this gap in our knowledge

## Methods

### Antibodies

Antibodies used were: anti-CCN2 (1:500; sc14939; Santa Cruz), anti-NOX4 (1:1000; ab133303, Abcam), anti-αSMA (1:2500, A5228, Sigma-Aldrich) and anti-β-actin (1:8000; A1978, Sigma-Aldrich). Horseradish peroxidase-conjugated donkey anti-goat (705-036-147), donkey anti-rabbit (711-036-152) and donkey anti-mouse (715-035-150) were from Jackson Immunoresearch Laboratories. AlexaFluor^™^-conjugated phalloidin (1:1000; PHDR1) was from Cytoskeleton.

### Cell culture

Human dermal (ATCC) and gingival (a generous gift from Dr. Douglas Hamilton, Western University) [14] fibroblasts from healthy humans (one cell line each was used in our experiments) were cultured in high glucose DMEM (Invitrogen, Burlington, Ontario) at 37°C in a humidified atmosphere containing 5% (v/v) CO_2_. All media was supplemented with 10% (v/v) FBS and 1% (v/v) antibiotic/antimycotic solution. Cells were seeded onto six-well cell culture dishes at a density of 6x10^5^ cells/well and were allowed to grow for 24 hours. Cells were then serum-starved in low glucose DMEM, 0.5% (v/v) FBS, for 16 hours, pre-treated for 45 minutes with either DMSO or one of the following inhibitors: N-acetylcysteine (NAC) (Calbiochem, 10 mM), diphenyleneiodonium (DPI) (Calbiochem, 10 μM), GKT-137831 (Cederlane, 30 μM), PF-573288 (Tocris, 10μM) or 5Z-7-Oxozeanol (Tocris, 400 nM) prior to the addition of TGFβ1 (R and D Systems, 4 ng/ml) for the durations indicated. SSc cells, used only in panels 5C and D, were obtained from patients as defined according to internationally agreed guidelines [15]. Patients included in the study were diffuse SSc (skin changes proximal to elbows and involving the trunk) within 2 years of the onset of skin changes. For panels 5C and D, SSc dermal fibroblasts were obtained from the forearm of patients with early onset diffuse scleroderma (systemic sclerosis, SSc); control fibroblasts (NF) were obtained from age- gender- and site-matched healthy individuals. For panels 5C and D, cells from three individuals with SSc and three healthy individuals were used. Cells were acquired under informed consent with the approval of the institutional review board of the Royal Free Hospital, and were isolated by explant culture from 4mm punch biopsies and were subsequently similarly cultured and treated.

### Real time RT-PCR

Experiments were conducted essentially as previously described [14]. Total RNA was harvested from cultured cells using phenol-chloroform extraction method and used for Real-Time(RT)-PCR. RNA concentration and integrity were determined using a Nanodrop 2000 (Thermo Scientific). RNA (40 ng/sample) was reverse transcribed and amplified using TaqMan Human Gene Expression assays (Applied Biosystems) in a 15 μl reaction containing qScript^™^ XLT 1-Step RT-qPCR ToughMix (Quanta Biosciences), TaqMan Assays-on-demand human gene specific primers (Applied Biosystems), and 6-carboxyfluroscein-labeled TaqMan MGB probe (Applied Biosystems). An ABI Prism 7900 HT sequence detector (Perkin-Elmer-Cetus, Vaudreuil, QC) was used for detection and analysis of amplified signal according to manufacturer’s instructions. Samples were run in triplicate, and expression values were standardized to control values from 18S primers using the ΔΔCt method. Statistical analysis on at least 3 independent experiments was done using a one-way ANOVA and Tukey’s post-hoc test with GraphPad Prism software. Results are expressed as mean +/- SD.

### Western blot analysis

Proteins were harvested using radioimmunoprecipitation assay (RIPA) buffer (150 mM NaCl, 100 mM Tris-HCl pH 7.4, 1% NP40, 0.1% SDS, 5 mM EDTA, 1X protease inhibitor cocktail). Protein concentrations were determined using BCA protein assay kit (Thermo Fischer Scientific) as described by the manufacturer’s instructions. Equal amounts of cell lysate (50 μg) were resolved by SDS-PAGE using 5% (w/v) stacking and 10% (w/v) separating polyacrylamide gels, and then transferred to 0.2-μm nitrocellulose membranes (Biorad), which were then blocked for 1 hour in 5% (w/v) non-fat milk diluted in Tris-buffered saline (TBST, 100 mM Tris-HCL, pH 7.4) with 0.01% (v/v) Tween-20, and incubated for 16 hours at 4°C with the indicated primary antibodies. Membranes were washed thrice with TBST, 5 minutes per wash, followed by incubation with HRP-conjugated secondary antibodies (Jackson Immunoresearch) for 1 hour at room temperature. SuperSignal^™^ West Pico Chemiluminescent Substrate (Thermo Fischer Scientific) was added to the membranes and proteins were visualized using X-ray film (Kodak). The results shown in figures are representative western blots of 3–5 experiments (as indicated).

### Indirect immunofluorescence analysis

Human dermal fibroblasts cultured on glass coverslips (VWR) were fixed for 20 minutes at room temperature with 4% (w/v) paraformaldehyde (PFA, Sigma-Aldrich) in PBS, washed twice with PBS, and then blocked with 10% (v/v) donkey serum and 0.1% (v/v) Triton X-100 (Sigma-Aldrich) diluted in PBS for 45 minutes at room temperature. Samples were incubated with AlexaFluor-conjugated Phalloidin for 1 hour in an enclosed humidity chamber. Finally, samples were washed thrice with PBS, and the coverslips were mounted onto slides using VECTASHIELD Mounting Media (Vector Laboratories) containing DAPI stain to visualize DNA. Images were obtained with a Zeiss Axio Imager.M1 microscope, using Northern Eclipse software. Brightness and contrast were adjusted uniformly with Photoshop CC software. The results shown in figures are representative images of 3 independent experiments.

### Collagen gel contraction assay

NF and SSc fibroblasts were cultured within three dimensional collagen lattices (FPCLs) [11]. First, 24-well tissue culture plates were coated with 2% (w/v) BSA in PBS and incubated for 16 hours at 37°C. For FPCLs, either NF or SSc fibroblasts were mixed with a neutral collagen solution containing one part 0.2 M HEPES (pH 8), four parts collagen (Nutragen, 3mg/ml, Advanced Biomatrix), and five parts MCDB-104 medium (Sigma-Aldrich), and then added to the 24-well tissue culture plate. GKT-137831 (30 μM) was added to the indicated wells. After polymerization, gels were mechanically detached from wells. Contraction of the gels was quantified by loss of gel weight over a 24 hour time period.

## Results

### The antioxidant NAC reduces the ability of TGFβ1 to induce CCN2 mRNA expression in human dermal and gingival fibroblasts

**Fig 1:**
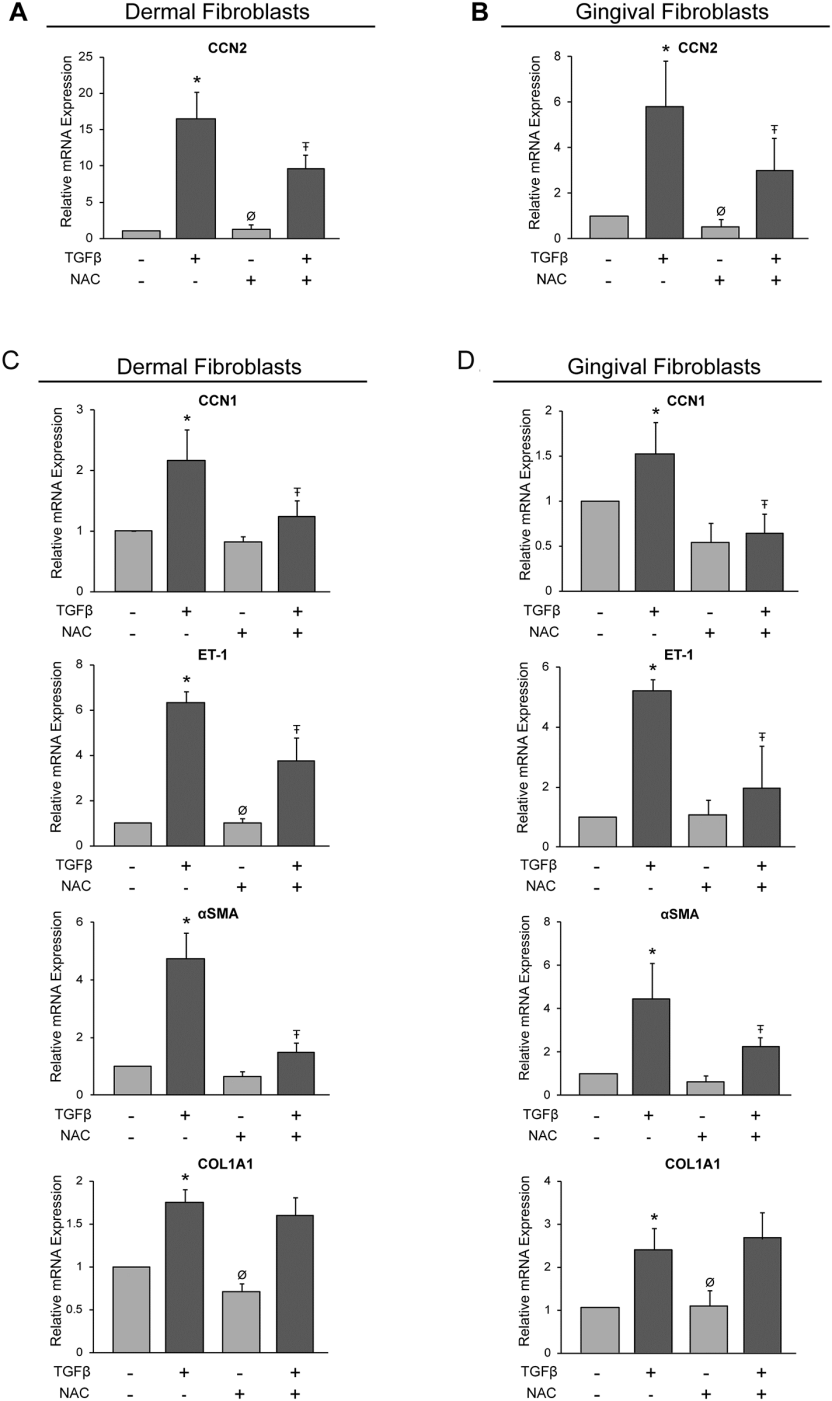
N-acetylcysteine reduces TGFβ1-induced mRNA expression in human dermal and gingival fibroblasts. Human dermal fibroblasts (A, C) and human gingival fibroblasts (B, D) were serum-starved overnight. Cells were incubated with the antioxidant, N-acetylcysteine (NAC, 10 mM), for 45 min followed by treatment with or without TGFβ1 (4ng/ml). (A, B) Total RNA was harvested 6 or, in the case of α-SMA or COL1A1 (see text), 24 hours later and subjected to RT-qPCR analysis for CCN2. Each sample was conducted in triplicate and CCN2 gene expression was normalized to 18S (internal control) using the ΔΔCt method. **(C, D)** RNA was harvested 6 or in the case of α-SMA or COL1A1 (see text), 24 hours later and subjected to RT-qPCR analysis for the indicated profibrotic genes. Each sample was conducted in triplicate and gene expression was normalized to 18S (internal control) using the ΔΔCt method. Results are expressed as a mean +/- SD (n = 4). One-Way ANOVA with post-hoc Tukey test was conducted. * = p<0.05 relative to control, Ŧ = p<0.05 relative to TGFβ, Ø = p<0.05 relative to NAC+TGFβ.

We first ascertained whether the antioxidant NAC reduced TGFβ-induced gene expression in dermal and gingival fibroblasts. Accordingly, we cultured dermal (HDFs) or gingival fibroblasts (HGFs) for 24 hours in 0.5% serum. Cells were then treated for 45 minutes with or without NAC, and cultured for an additional 6 hours in the presence or absence of TGFβ1. RNA was then extracted and subjected to real time PCR analysis. One of the key mediators of fibrosis is the profibrotic marker, CCN2 [4]. As this mRNA is potently induced by TGFβ [4, 14, 16], we first evaluated whether the ability of TGFβ1 to induce this transcript in HDFs and HGFs was sensitive to NAC. Compared with 18S mRNA expression in the presence of DMSO, TGFβ induced CCN2 mRNA expression in both cell types at 6 h post-TGFβ1 addition in a NAC-sensitive fashion, suggesting ROS was required for this phenomenon (Fig 1A and 1B). NAC did not appreciably reduce basal CCN2 mRNA expression, indicating that ROS did not contribute to basal CCN2 mRNA expression in these cells (Fig 1A and 1B).

### NAC reduces TGFβ1-induced CCN1, ET-1 and α-SMA mRNA expression in human dermal and gingival fibroblasts

To further examine whether, in principle, NAC could be used to block profibrotic responses to TGFβ in both dermal and gingival fibroblasts, we next tested whether NAC could suppress the ability of TGFβ to induce the mRNA expression of other pro-fibrotic genes. CCN1, a member of the CCN family of matricellular proteins, has similar *in vitro* functions to CCN2 [16] and is associated with lung fibrosis [17]. Endothelin-1 (ET-1) is a vasoconstricting peptide and, as a downstream mediator of profibrotic TGFβ signaling has been implicated in the fibroproliferative phenotype [4]. The protein α-smooth muscle actin (α-SMA) is a marker of activated fibroblasts, and plays a role in wound closure and fibrosis [18]. Increased expression of ECM components, such as type I collagen, are hallmarks of fibrosis. In both HDF and HGF, statistically significant TGFβ1-induced CCN1 and ET-1 expression was apparent at 6 h post-addition of TGFβ and treatment with NAC significantly reduced their induction (Fig 1C and 1D). For both COL1A1 and α-SMA, a 24 h time point was examined as TGFβ is unable to significantly induce these transcripts 6 h post-addition (data not shown). The ability of TGFβ to induce αSMA in HDF and HGF was sensitive to NAC treatment; however, induction of COL1A1 mRNA was unaffected in both cell types (Fig 1C and 1D). Accordingly, expression of CCN2, CCN1, ET-1 and α-SMA were selected for further analysis.

### The selective NOX inhibitor DPI reduces TGFβ1-induced CCN2, CCN1, ET-1, and α-SMA mRNA expression in human dermal and gingival fibroblasts

**Fig 2:**
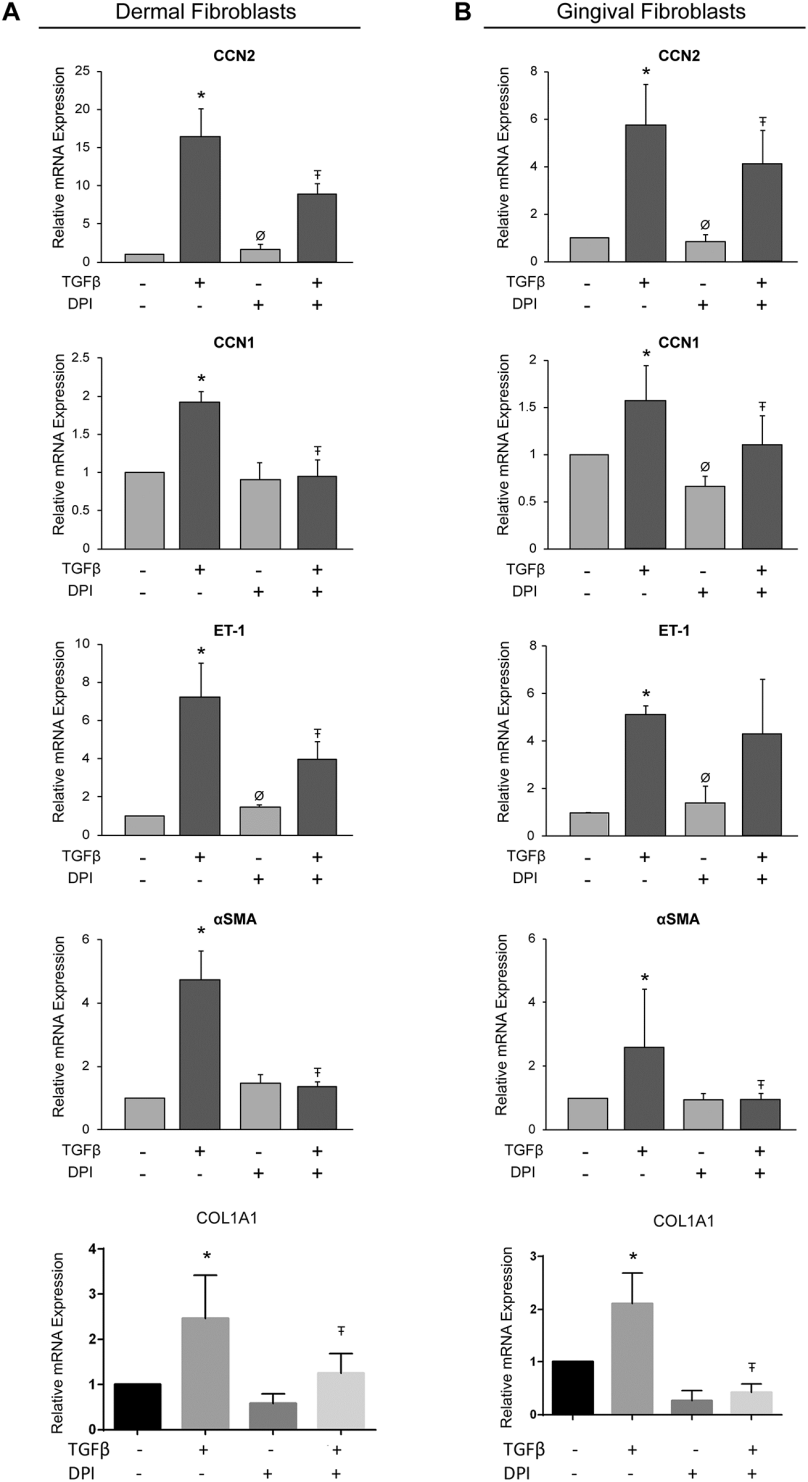
Diphenyleneiodonium reduces TGFβ1-induced mRNA expression of pro-fibrotic genes in human dermal and gingival fibroblasts. Human dermal fibroblasts (A) and human gingival fibroblasts (B) were serum-starved overnight. Cells were incubated with the broad-range NOX protein inhibitor, diphenyleneiodonium (DPI, 10 μM), for 45 min followed by treatment with or without TGFβ1 (4ng/ml). Total RNA was harvested either 6 or 24 (in the case of α-SMA, see text) hours later and subjected to RT-qPCR analysis for the indicated genes. Each sample was conducted in triplicate and gene expression was normalized to 18S (internal control). Results are expressed as a mean +/- SD (n = 4). One-Way ANOVA with post-hoc Tukey test was conducted. * = p<0.05 relative to control, Ŧ = p<0.05 relative to TGFβ1, Ø = p<0.05 relative to DPI+TGFβ1.

Diphenyleneiodonium (DPI) inhibits the activity of the flavoenzymes, NOX enzymes and dual oxidases, which are seven specific O_2_^.−^ and H_2_O_2_ generating enzymes [19]. Consistent with the notion that NOX enzymes are required for the induction of a subset of fibrogenic responses in fibroblasts, we found that DPI impaired the ability of TGFβ1 to induce CCN2, CCN1 and α-SMA mRNA expression in both HDF and HGF (Fig 2A and 2B). However, DPI was found to statistically significantly impair ET-1 induction in dermal but not gingival fibroblasts (Fig 2A and 2B) In spite of this latter apparent difference, NOX enzymes seemed to mediate TGFβ1-induced profibrotic mRNA expression in both HGF and HDF. Please note that DPI also reduced TGFβ1-induced COL1A1 mRNA expression in both cell types (Fig 2A and 2B, see Discussion).

**Fig 3:**
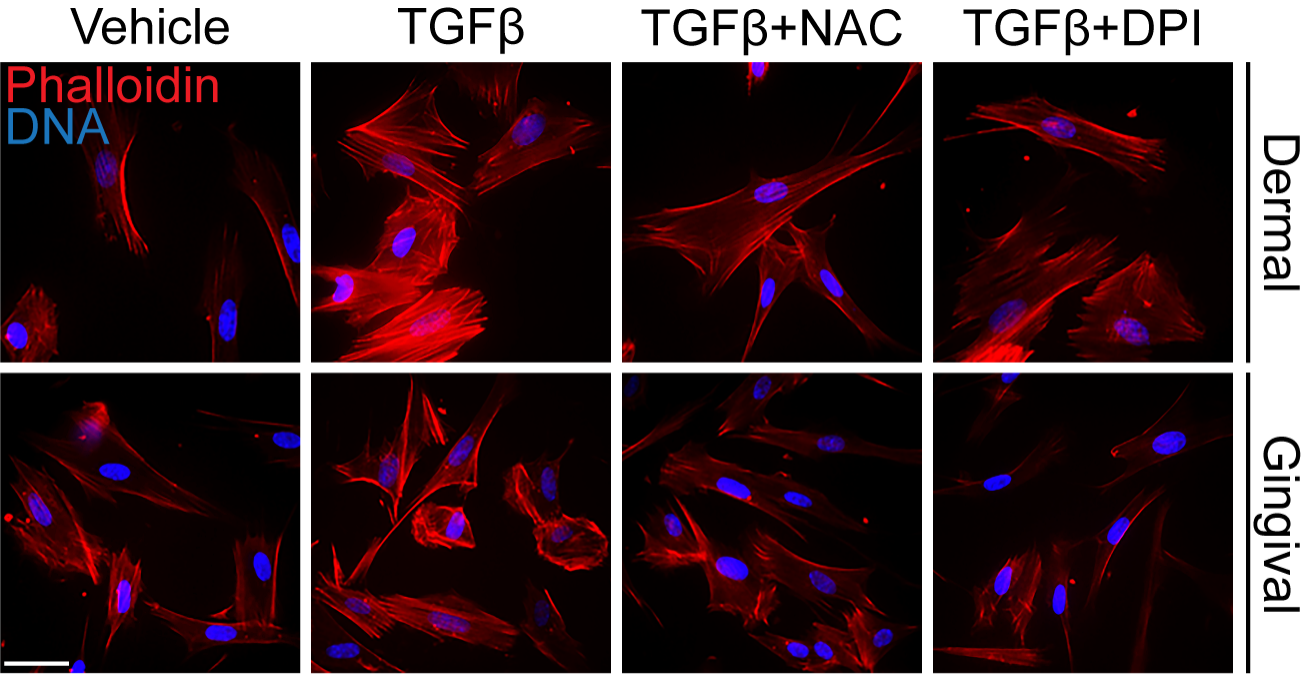
N-acetylcysteine and diphenyleneiodonium reduce TGFβ-induced stress fiber formation in human dermal and gingival fibroblasts. A) Human dermal fibroblasts and human gingival fibroblasts were serum-starved overnight. Cells were incubated with either N-acetylcysteine (10 mM) or diphenyleneiodonium (10 μM) for 45 min followed by treatment with or without TGFβ1 (4ng/ml). Cells were fixed with paraformaldehyde 24 hours later, and stress fiber formation was detected using AlexaFluor-conjugated Phalloidin. DAPI was used to visualize DNA. Representative images are shown (n = 3); bar, 25 μm. Note that, as previously described [19–21], actin-containing stress fibers were not as readily apparent in HDF.

As CCN2, αSMA and ET-1 are all markers of actin stress-fiber containing myofibroblasts, we next investigated whether NAC and DPI could potentially block the ability of TGFβ1 to induce actin-containing stress fibers in dermal and gingival fibroblasts. Accordingly, HDF and HGF were cultured with or without TGFβ1 for 24 hours in the presence or absence of either DPI or NAC, and then processed for immunofluorescence microscopy using rhodamine-phalloidin stain to detect the overall appearance of actin-containing stress fibers. We found that treatment of HDF with TGFβ1 caused formation of actin-containing stress fibers, in a fashion sensitive to DPI and NAC (Fig 3). TGFβ1 caused an appreciable increase in rhodamine-phalloidin signal in HGF; however, consistent with previous observations [20–22], actin-containing stress fibers were not readily apparent. Nonetheless, this modest increase in rhodamine-phalloidin signal was sensitive to NAC and DPI (Fig 3). These data are consistent with the notion that NAC and DPI block the ability of TGFβ1 to activate fibroblasts.

### TGFβ1-induced CCN2 and α-SMA protein expression is reduced by NAC, DPI and the NOX1/4 inbibitor GKT-137831 in human dermal and gingival fibroblasts

**Fig 4:**
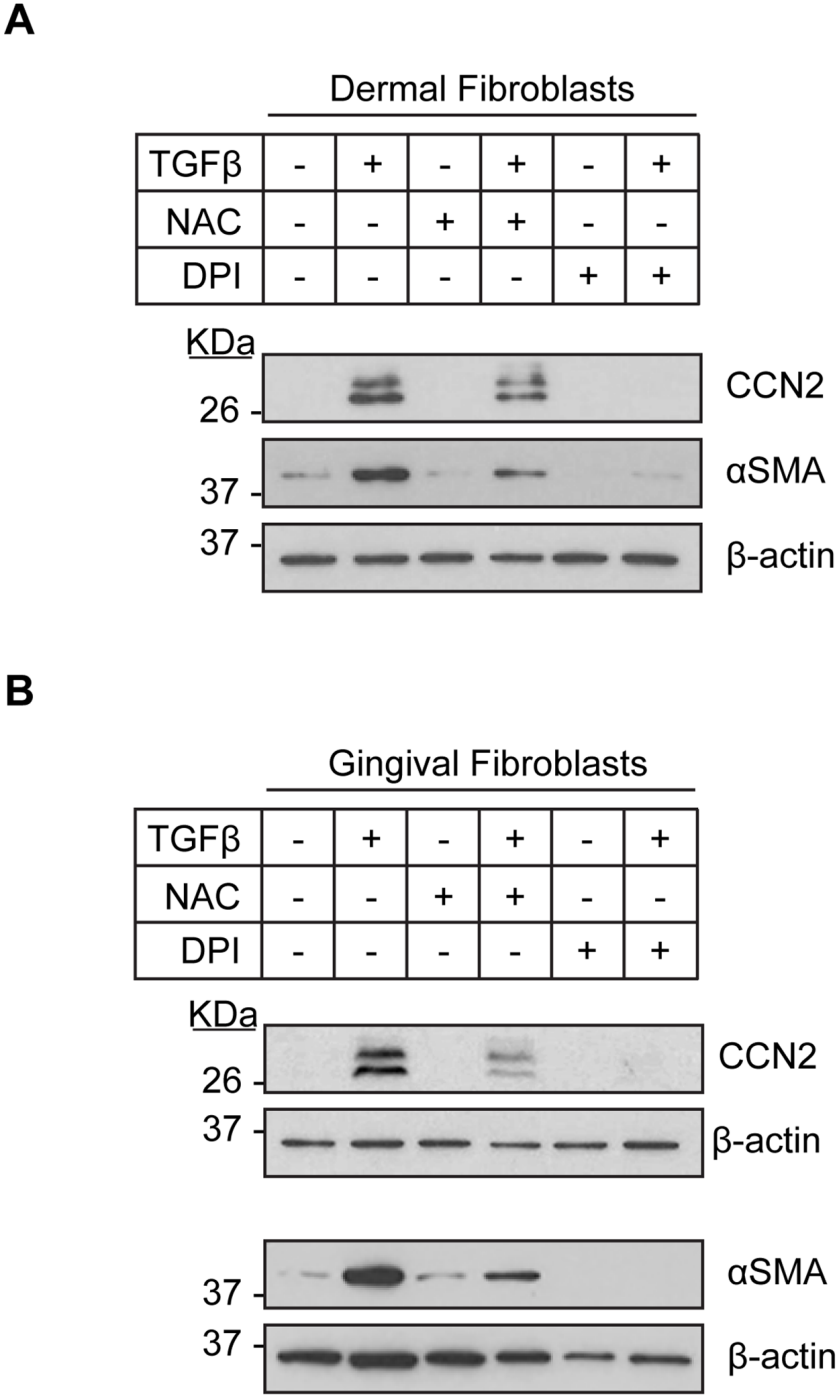
TGFβ1-induced CCN2 and αSMA protein expression in human dermal and gingival fibroblasts is reduced by N-acetylcysteine and diphenyleneiodonium. Human dermal fibroblasts (A) and human gingival fibroblasts (B) were serum-starved overnight. Cells were incubated with either N-acetylcysteine (10 mM) or diphenyleneiodonium (10 μM) for 45 min followed by treatment with or without TGFβ1 (4ng/ml) for 24 hours. Protein lysates were prepared and subjected to western blot analysis with the indicated antibodies. β-actin was used to normalize for protein loading. Representative western blots are shown (n = 3).

To further investigate the involvement of antioxidants and NOX inhibition to impair fibrogenic responses to TGFβ, we focused on CCN2 and α-SMA as these are key markers of activated, fibrotic fibroblasts [4, 16, 18]. Thus, we assessed whether NAC or DPI affected TGFβ1-induced CCN2 and α-SMA protein expression in HDF and HGF. To perform this analysis, we cultured HDF and HGF for 24 hours in 0.5% serum. Cells were then treated for 45 minutes with or without NAC or DPI, and cultured for an additional 24 hours in the presence or absence of TGFβ1. As anticipated, TGFβ induced CCN2 and α-SMA protein in both cell-types in the presence of DMSO (Fig 4A and 4B). However, addition of NAC or DPI blocked the ability of TGFβ to induce CCN2 and α-SMA protein expression (Fig 4A and 4B).

**Fig 5:**
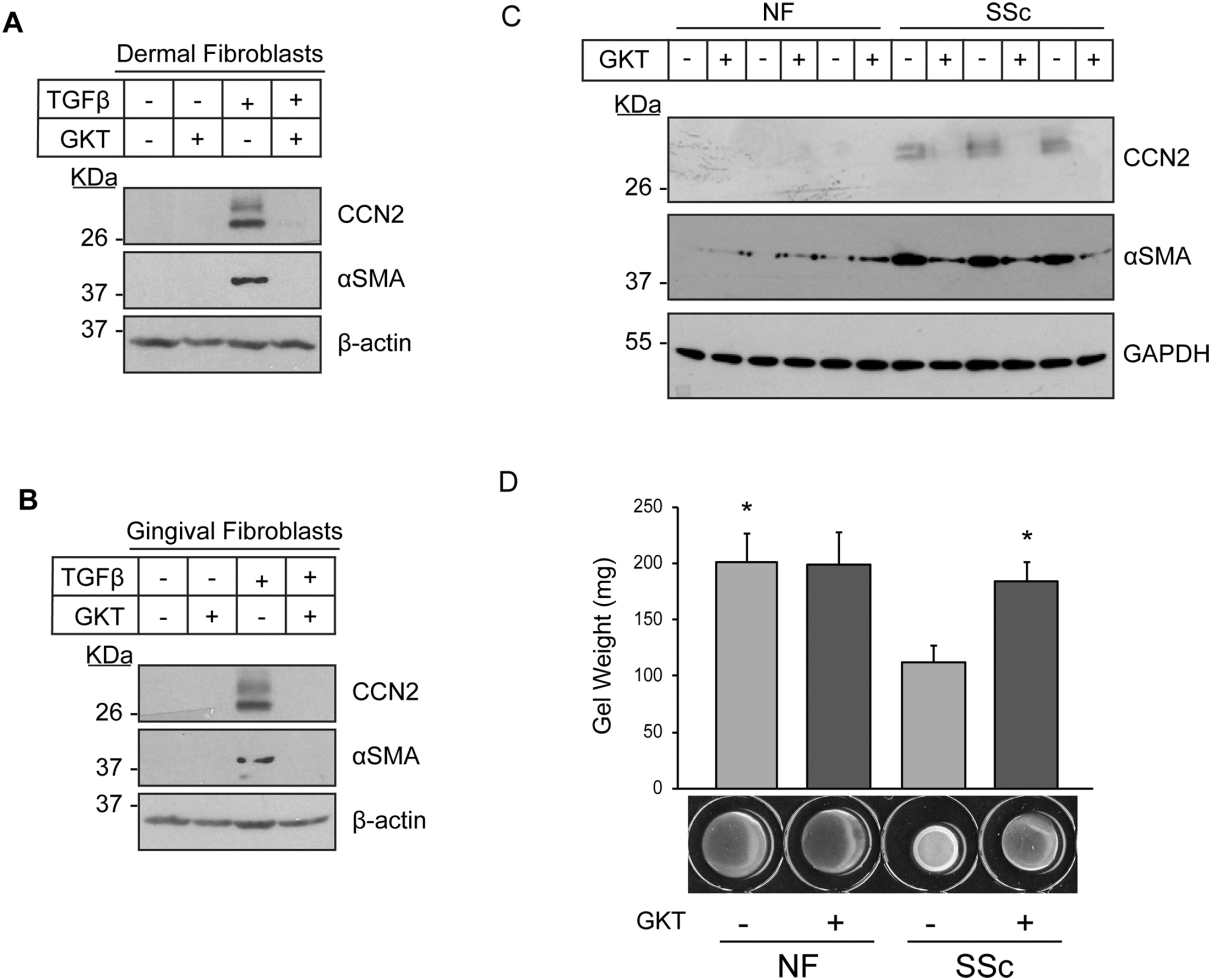
Inhibition of NOX4 reduces TGFβ1-induced CCN2 and αSMA protein expression in human dermal and gingival fibroblasts and the overexpression of CCN2 and α-SMA, as well as the contractility of lesional SSc dermal fibroblasts. Human dermal fibroblasts (A) and human gingival fibroblasts (B) were serum-starved overnight. Cells were incubated with GKT-137831 (30 μM) for 45 min followed by treatment with or without TGFβ1 (4ng/ml) for 24 hours. Protein lysates were prepared and subjected to western blot analysis with the indicated antibodies. β-actin was used to normalize for protein loading. Representative western blots are shown (n = 3). **(C)** Dermal fibroblasts cultured from healthy individuals (NF, normal fibroblasts) and those with scleroderma (systemic sclerosis, SSc) were serum-starved overnight. Cells were incubated with GKT-137831 (30μM) for 24 hours. Protein lysates were prepared and subjected to western blot analysis with the indicated antibodies. GAPDH was used to normalize for protein loading. Representative western blots containing lysates from three different patients are shown (n = 3). (D) NF and SSc fibroblasts were cultured within 3D-collagen lattices in the presence and absence of GKT-137831 (30 μM). After polymerization, the gels were mechanically detached from the wells, and the contraction of the gel was quantified. Results are expressed as a mean +/- SD (n = 3). One-Way ANOVA with post-hoc Tukey test was conducted. * = p<0.05 relative to SSc (-) GKT.

To further probe the involvement of NOX enzymes in the induction of fibrogenic responses in fibroblasts, we tested the ability of the NOX1/4 inbibitor GKT-137831 to block TGFβ1-induced CCN2 and α-SMA protein expression in HDF and HGF. We chose this inhibitor as NOX4 is highly expressed in dermal fibroblasts (S1 Fig) and also upgregulated in SSc fibroblasts [12]. Furthermore, of the 5 NOX enzymes, only NOX4 mRNA was induced by TGFβ1 in HDF (S1 Fig). Importantly, NOX1 mRNA was not detected in HDF suggesting that the effects of GKT-137831 are likely to be due primarily to the contribution of NOX4 (S1 Fig). Paralleling our data with NAC and DPI, we found that addition of GKT-137831 impaired the ability of TGFβ1 to induce CCN2 and α-SMA protein expression in both HDF and HGF (Fig 5A and 5B).

To extend our studies and to provide a potential translational context, we assessed the ability of GKT-137831 to modify the persistent fibrotic phenotype of lesional SSc dermal fibroblasts. For these studies (Fig 5C and 5D) cells from three individuals with SSc and three healthy individuals were used. Similarly, GKT-137831 blocked the overexpression of CCN2 and α-SMA in lesional SSc dermal fibroblasts, but did not have an effect on normal healthy adult fibroblasts (NF) (Fig 5C). Moreover, GKT-137831 also impaired the enhanced ability of SSc dermal fibroblasts to contract a collagen gel matrix but did not appreciably affect the contractile ability of normal healthy adult fibroblasts (NF) (Fig 5D).

Previously, we showed that TGFβ-induced CCN2 expression in HDF and HFF was blocked by focal adhesion kinase (FAK) or TGFβ-activated kinase 1 (TAK1) inhibition [8,11,23,24]. To further explore the underlying involvement of NOX enzymes in the induction of fibrogenic responses in fibroblasts, we assessed if the inhibition of FAK and TAK1 would potentially block TGFβ1-induced NOX4 expression. Confirming prior data, in HDF and HGF, TGFβ1-induced CCN2 mRNA expression was impaired by FAK or TAK1 inhibition (S2A and S2B Fig). Moreover, TGFβ1-induced NOX4 mRNA expression was impaired by FAK and TAK1 inhibition. Similar results were obtained when the effects of FAK and TAK1 inhibition on TGFβ1-induced NOX4 protein expression was examined (S3 and S4 Figs). Collectively, our results are consistent with the notion that antioxidants or the NOX1/4 inhibitor GKT-137831 might be used as an antifibrotic agent.

## Discussion

Pathological scarring is caused by the myofibroblast, a type of fibroblast containing contractile α-SMA stress fibers [3, 18]. Fibrotic responses in the oral cavity are hyperproliferative, resulting in gingival overgrowths instead of scar tissue that possess abundant myofibroblasts [4, 20–22]. In every model thus far examined, CCN2 expression is an excellent surrogate marker for the severity of fibrosis [25]. Moreover, emerging evidence supports the notion that CCN2 is a key fibrogenic mediator both in dermal and in gingival fibroblasts [4, 6, 26–28]. Intriguingly, CCN2 expression by fibroblasts is not required for normal tissue repair, suggesting that it may represent a specific antifibrotic target [29]. The potent fibrogenic cytokine TGFβ has been linked to both gingival and dermal fibrosis. TGFβ1 induces CCN2 expression in both dermal and gingival fibroblasts [6, 7, 30–33]; however, gingival fibroblasts appear less potent in their response to TGFβ, and this difference has been linked to reduced adhesive signaling or responses to mechanical strain in this cell type [8, 21, 34]. Similarly, in HGF, TGFβ1 or profibrotic signaling does not ultimately result in abundant stress fibers; i.e., the full-blown myofibroblast phenotype *in vitro* or *in vivo* [8, 20–22, 30]. Thus, investigating how TGFβ activates profibrotic gene expression in HGF and HDF is of inherent value.

In this report, we find that TGFβ1 can induce expression of profibrotic genes, including CCN2 and α-SMA, via ROS/NOX in normal dermal and gingival fibroblasts, as the antioxidant NAC and the NOX inhibitors DPI can block these process in both cell types. Consistent with these prior reports, in this study we find that the fold-increase of mRNA expression in response to TGFβ1, most notably that of CCN2, appears less in HGF then in HDF. Taken together, these data suggest that although HGF and HDF may differ somewhat in their responses to TGFβ1 it is not likely due to a differential involvement of the NOX/ROS pathway in the two cell types. However, it is intriguing to note that, unlike in HDF, TGFβ-induced ET-1 mRNA expression in HGF was not sensitive to DPI.

It is interesting to note that prior reports indicated, in fibroblasts, TAK1 and FAK mediate collagen expression including in response to TGFβ1 [11, 23, 24]. Moreover, NAC reduced type I collagen expression in SSc fibroblasts [11]; however, in this study NAC did not appreciably affect TGFβ1-induced COL1A1 expression. These results may reflect that type I collagen is highly regulated post-transcriptionally and only modest induction of type I collagen mRNA by TGFβ1 is observed (see reference [35] and references therein). It is interesting to note that DPI blocked TGFβ1-induced COL1A1 expression in both cell types (Fig 2); please note thar DPI has other effects on cells other than NOX inhibition and is known to have unrelated side effects (see reference [36] and references therein). It should also be pointed out that adhesive signaling (FAK) operates independent of TGFβ pathways as it is activated by a wide variety of signaling pathways, although there is an obligate requirement for adhesion in terms of TGFβ-induced myofibroblast differentiation [37, 38]. Indeed, FAK inhibition can lower gene expression (eg. NOX4, S3 Fig) in the absence of exogenous TGFβ1.

Antioxidants have been suggested as potential treatments for fibrosis; high-dose NAC given for one year with prednisone and azathioprine, significantly slows down disease progression in idiopathic pulmonary fibrosis, and in lungs of SSc and Chronic Obstructive Pulmonary Disease patients [10, 39–41]. NAC has been used in the clinical practice for many decades and is readily available as a dietary supplement [21] and therefore might represent an ideal strategy to control fibrogenesis in general. Moreover, the selective NOX1/4 inhibitor GKT-137831 is under clinical development [13]. Indeed, data we report herein, including our observations that GKT-137831 can reduce CCN2 and α-SMA expression and collagen gel contraction in SSc fibroblasts, suggest that future experiments aimed at assessing whether antioxidants or NOX inhibitors could be used to suppress the fibrotic phenotype are justified.

## Supporting information

S1 Fig NOX4 mRNA is highly expressed in human dermal fibroblasts. A) Total RNA was harvested from cultured human dermal fibroblasts and subjected to TaqMan RT-qPCR analysis for the indicated NOX proteins. Each sample was conducted in triplicate and 18S RNA was used as an internal control. NOX4 was set to 1 and the expression of each NOX protein was compared. Results are expressed as a mean +/- SD (n = 4). * = p<0.05 (unpaired Student T-Test) relative to NOX4. Abbreviations: ND, not detected. B) Human dermal fibroblasts were serum-starved overnight, followed by a 6 hour incubation with or without TGFβ1 (4ng/ml). Total RNA was harvested and subjected to TaqMan RT-qPCR analysis for the indicated NOX proteins. Each sample was conducted in triplicate and 18S RNA was used as an internal control. Results are expressed as a mean +/- SD (n = 4). * = p<0.05 (unpaired Student T-Test) relative to the no TGFβ1 treatment (–). Abbreviations: ND, not detected.
(TIF)

S2 Fig Inhibition of FAK and TAK1 reduces TGFβ-induced CCN2 and NOX4 mRNA expression in human dermal and gingival fibroblasts. Human dermal fibroblasts (A) and human gingival fibroblasts (B) were serum-starved overnight. Cells were incubated with either PF-573288 (10μM) or 5Z-7-Oxozeanol (400 nM) for 45 min followed by treatment with or without TGFβ1 (4ng/ml). Total RNA was harvested 6 hours later and subjected to TaqMan RT-qPCR analysis for CCN2 and NOX4 RNA expression. Each sample was conducted in triplicate and 18S RNA was used as an internal control. Results are expressed as a mean +/- SD (n = 5, PF-573288; n = 3, 5Z-7-Oxozeanol). One-Way ANOVA with post-hoc Tukey test was conducted. * = p<0.05 relative to control, Ŧ = p<0.05 relative to TGFβ, Ø = p<0.05 relative to PF+TGFβ or OXO+TGFβ.
(TIF)

S3 Fig Inhibition of FAK reduces TGFβ-induced CCN2 and NOX4 protein expression in human dermal and gingival fibroblasts. Human dermal fibroblasts (A) and human gingival fibroblasts (B) were serum-starved overnight. Cells were incubated with PF-573288 (10μM) for 45 min followed by treatment with or without TGFβ1 (4ng/ml) for 24 hours. Protein lysates were prepared and subjected to western blot analysis with the indicated antibodies. β-actin was used to normalize for protein loading. Representative western blots are shown (n = 3).
(PDF)

S4 Fig Inhibition of TAK1 reduces TGFβ-induced CCN2 and NOX4 protein expression in human dermal and gingival fibroblasts. Human dermal fibroblasts (A) and human gingival fibroblasts (B) were serum-starved overnight. Cells were incubated with 5Z-7-Oxozeanol (400–1600 nM) for 45 min followed by treatment with or without TGFβ1 (4ng/ml) for 24 hours. Protein lysates were prepared and subjected to western blot analysis with the indicated antibodies. β-actin was used to normalize for protein loading. Representative western blots are shown (n = 3).
(PDF)
