# Supplementary figures and images for: Antioxidants and NOX1/NOX4 inhibition blocks TGFβ1-induced CCN2 and α-SMA expression in dermal and gingival fibroblasts
Source: PLoS One. 2017 Oct 19;12(10):e0186740. doi: 10.1371/journal.pone.0186740 (PMC5648211; doi:10.1371/journal.pone.0186740)

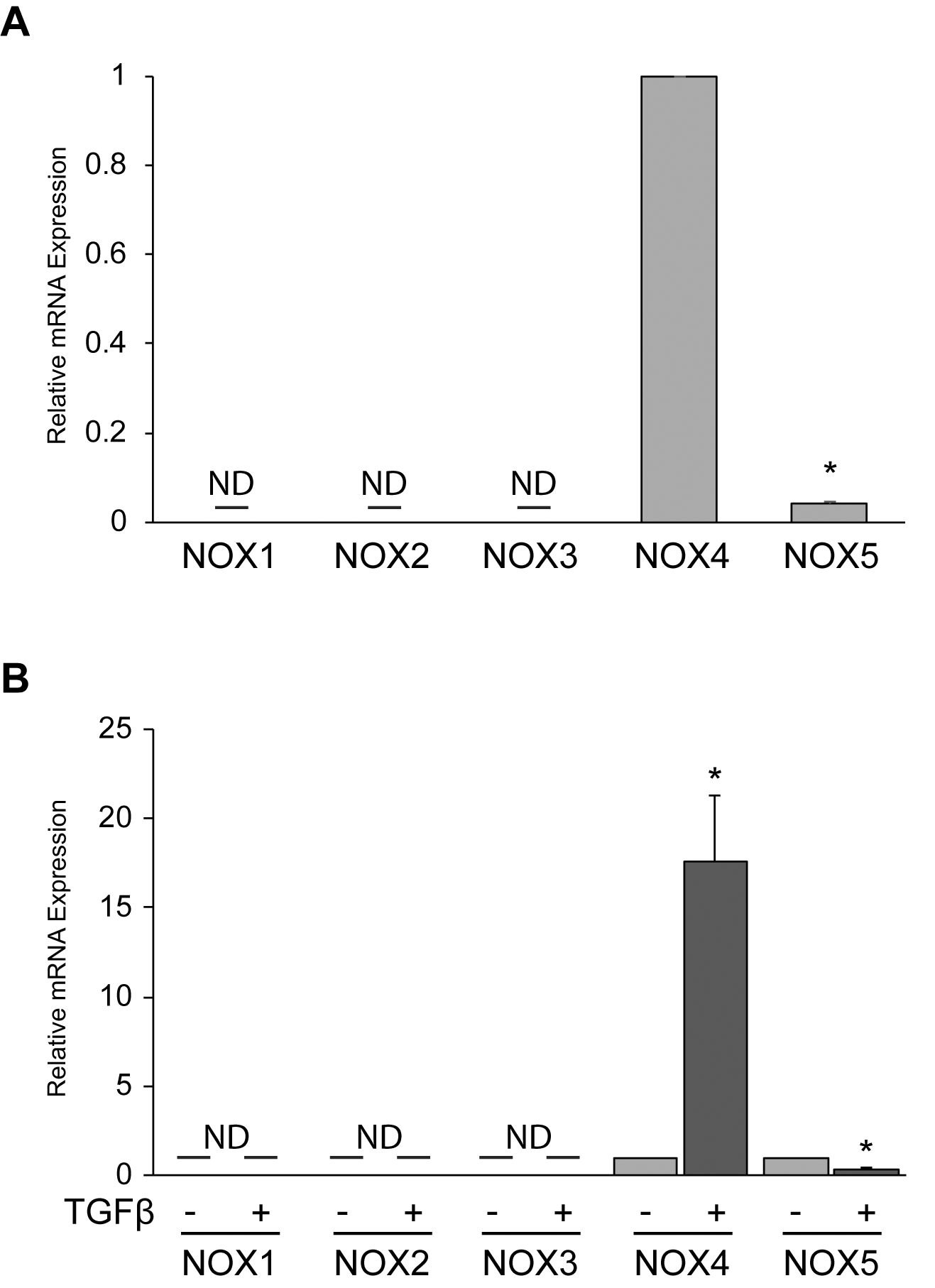

Supplement: S1 Fig — A) Total RNA was harvested from cultured human dermal fibroblasts and subjected to TaqMan RT-qPCR analysis for the indicated NOX proteins. Each sample was conducted in triplicate and 18S RNA was used as an internal control. NOX4 was set to 1 and the expression of each NOX protein was compared. Results are expressed as a mean +/- SD (n = 4). * = p<0.05 (unpaired Student T-Test) relative to NOX4. Abbreviations: ND, not detected. B) Human dermal fibroblasts were serum-starved overnight, followed by a 6 hour incubation with or without TGFβ1 (4ng/ml). Total RNA was harvested and subjected to TaqMan RT-qPCR analysis for the indicated NOX proteins. Each sample was conducted in triplicate and 18S RNA was used as an internal control. Results are expressed as a mean +/- SD (n = 4). * = p<0.05 (unpaired Student T-Test) relative to the no TGFβ1 treatment (–). Abbreviations: ND, not detected. (TIF) [file pone.0186740.s001.tif]

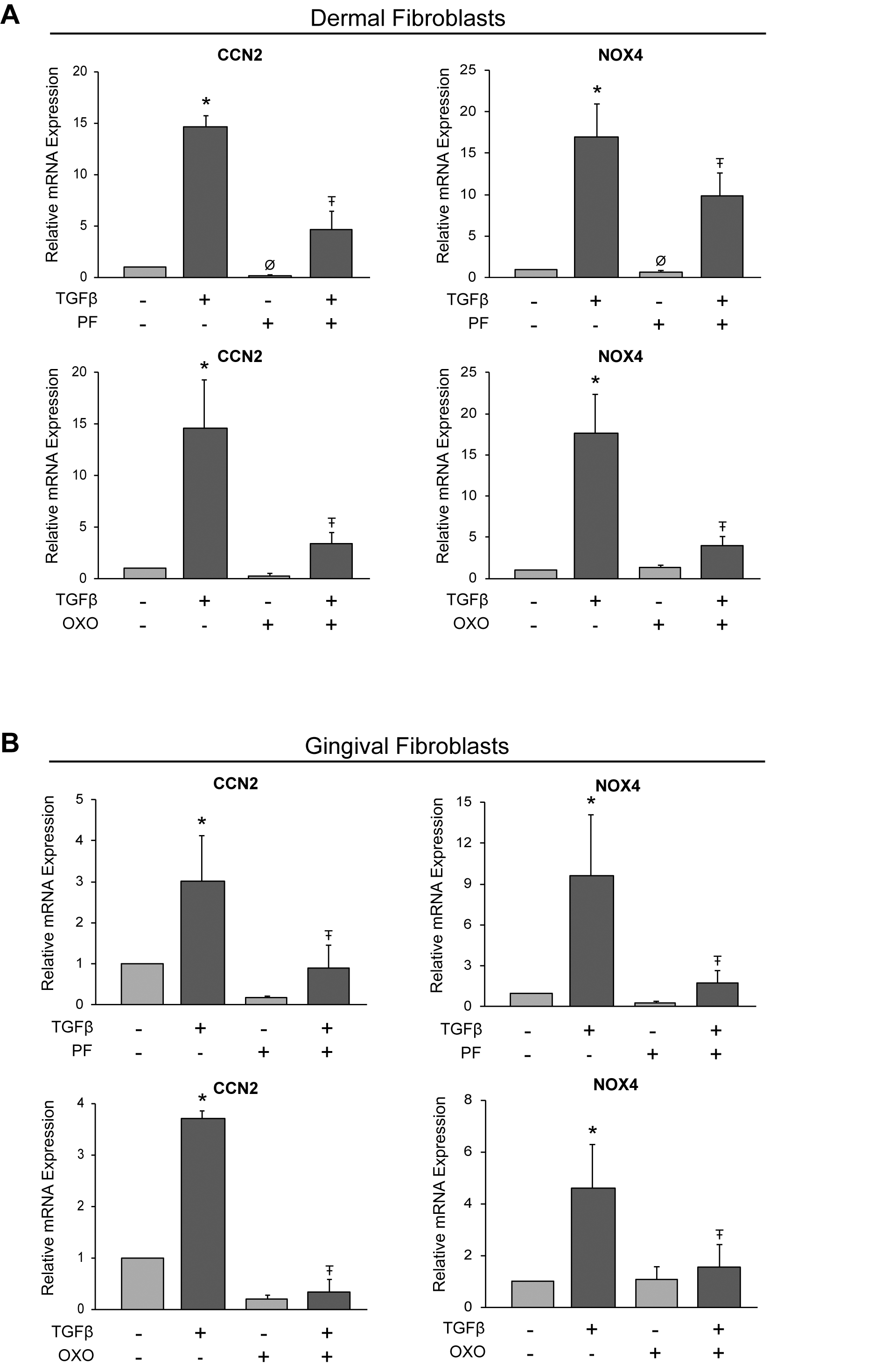

Supplement: S2 Fig — Human dermal fibroblasts (A) and human gingival fibroblasts (B) were serum-starved overnight. Cells were incubated with either PF-573288 (10μM) or 5Z-7-Oxozeanol (400 nM) for 45 min followed by treatment with or without TGFβ1 (4ng/ml). Total RNA was harvested 6 hours later and subjected to TaqMan RT-qPCR analysis for CCN2 and NOX4 RNA expression. Each sample was conducted in triplicate and 18S RNA was used as an internal control. Results are expressed as a mean +/- SD (n = 5, PF-573288; n = 3, 5Z-7-Oxozeanol). One-Way ANOVA with post-hoc Tukey test was conducted. * = p<0.05 relative to control, Ŧ = p<0.05 relative to TGFβ, Ø = p<0.05 relative to PF+TGFβ or OXO+TGFβ. (TIF) [file pone.0186740.s002.tif]

**A**Dermal Fibroblasts

|             |   |   |   |   |
|-------------|---|---|---|---|
| TGF $\beta$ | - | - | + | + |
| PF          | - | + | - | + |

KDa

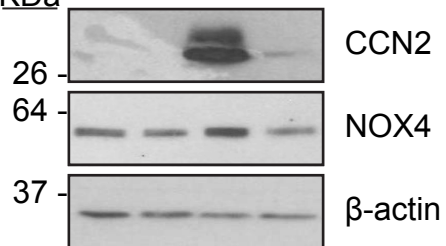**B**Gingival Fibroblasts

|             |   |   |   |   |
|-------------|---|---|---|---|
| TGF $\beta$ | - | - | + | + |
| PF          | - | + | - | + |

KDa

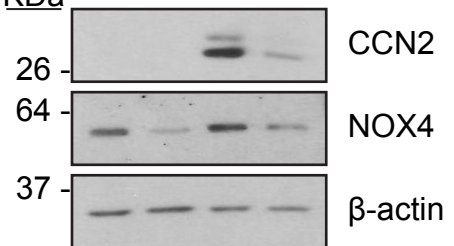

Supplement: S3 Fig — Human dermal fibroblasts (A) and human gingival fibroblasts (B) were serum-starved overnight. Cells were incubated with PF-573288 (10μM) for 45 min followed by treatment with or without TGFβ1 (4ng/ml) for 24 hours. Protein lysates were prepared and subjected to western blot analysis with the indicated antibodies. β-actin was used to normalize for protein loading. Representative western blots are shown (n = 3). (PDF) [file pone.0186740.s003.pdf]

**A**

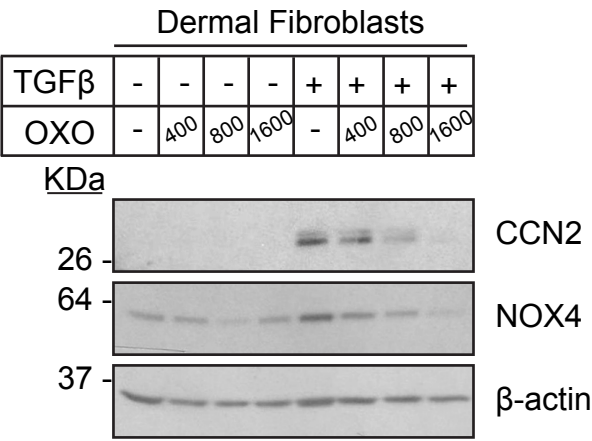

**B**

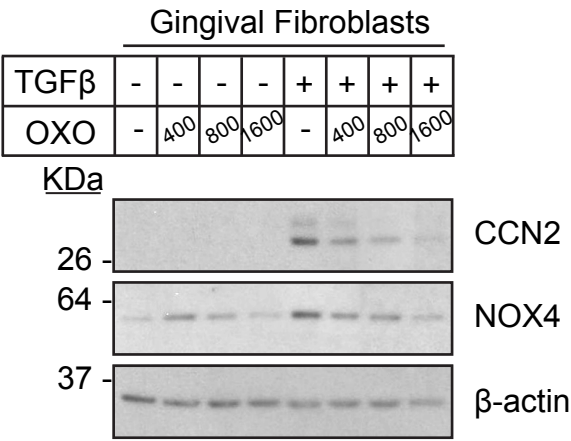

Supplement: S4 Fig — Human dermal fibroblasts (A) and human gingival fibroblasts (B) were serum-starved overnight. Cells were incubated with 5Z-7-Oxozeanol (400–1600 nM) for 45 min followed by treatment with or without TGFβ1 (4ng/ml) for 24 hours. Protein lysates were prepared and subjected to western blot analysis with the indicated antibodies. β-actin was used to normalize for protein loading. Representative western blots are shown (n = 3). (PDF) [file pone.0186740.s004.pdf]
